# Inhibition of Complex I of the Respiratory Chain, but Not Complex III, Attenuates Degranulation and Cytokine Secretion in Human Skin Mast Cells

**DOI:** 10.3390/ijms231911591

**Published:** 2022-09-30

**Authors:** Thomas Buttgereit, Moritz Pfeiffenberger, Stefan Frischbutter, Pierre-Louis Krauß, Yuling Chen, Marcus Maurer, Frank Buttgereit, Timo Gaber

**Affiliations:** 1 Institute of Allergology, Charité—Universitätsmedizin Berlin, Corporate Member of Freie Universität Berlin and Humboldt-Universität zu Berlin, 12203 Berlin, Germany; 2 Fraunhofer Institute for Translational Medicine and Pharmacology ITMP, Allergology and Immunology, 12203 Berlin, Germany; 3 Department of Rheumatology and Clinical Immunology, Charité—Universitätsmedizin Berlin, Corporate Member of Freie Universität Berlin and Humboldt-Universität zu Berlin, 10117 Berlin, Germany; 4 German Rheumatism Research Centre (DRFZ) Berlin, a Leibniz Institute, 10117 Berlin, Germany

**Keywords:** human skin mast cell, mitochondria, degranulation, myxothiazol, rotenone A

## Abstract

The mechanisms of mast cell (MC) degranulation and MC-driven skin symptoms are well-described. In contrast, data about the role of mitochondrial respiration for immune functions of human skin MCs are lacking. Oxygen consumption rate (OCR) in primary human skin MCs during IgE-mediated activation in the absence of glucose was examined using a metabolic flux analyzer. Effects of the inhibition of mitochondrial complex I (by rotenone A) and III (by myxothiazol) on degranulation and cytokine secretion (IL-4, IL-5, IL-6, IL-13, TNF-α, and GM-CSF) were explored by the β-hexosaminidase release assay and multiplex ELISA. IgE-mediated activation rapidly increased the mitochondrial OCR and extracellular acidification; the contribution of non-mitochondrial oxygen consumption remained unchanged at lower levels. Both myxothiazol and rotenone A reduced OCR, the mitochondrial parameters, and extracellular acidification; however, myxothiazol did not affect degranulation and cytokine secretion. In contrast, degranulation and the secretion of IL-6, IL-13, TNF-α, and GM-CSF were reduced by rotenone A, whereas the secretion of IL-4 and IL-5 was not significantly affected. The inhibitors did not affect cell viability. Our results highlight the important role played by mitochondrial respiration in primary human skin MCs and allow for a conclusion on a hierarchy of their effector functions. Drugs targeting specific pathways in mitochondria may provide future options to control MC-driven skin symptoms.

## 1. Introduction

Mast cells (MCs) are resident proinflammatory cells in the mucosal and connective tissue that orchestrate a large array of innate and adaptive immune responses [1]. They are the key driver of allergic reactions and diseases, where MC activation is caused by cross-linkage of the high-affinity IgE receptor (FcεRI) by allergens and pre-bound immunoglobulin (Ig) E [2]. IgE-mediated activation of MCs initiates a multiphasic reaction with the early release of preformed granules (degranulation) containing histamine, heparin, and proteases (e.g., tryptase). This is followed by the de novo production and release of lipid mediators including prostaglandins, leukotrienes, and platelet-activating factor as well as several cytokines and chemokines [3] In addition to their pivotal role in allergies and other diseases, MCs are considered to be involved in the protection from parasites and venoms [4,5].

Various skin conditions manifest with MC-mediated signs and symptoms. These include, for example, anaphylaxis [6], indolent systemic mastocytosis [7], and chronic urticaria [8,9]. In these diseases, cutaneous signs and symptoms such as wheals, angioedema, flush reactions, and pruritus are linked to and caused by skin MC activation. In contrast, the entity of idiopathic mast cell activation syndrome (MCAS) remains a subject of critical discussion [10,11,12].

Like other cells, MCs require energy for housekeeping functions, proliferation, differentiation, and activation [13,14]. Adequate adenosine triphosphate (ATP) levels are essential for MC functions [14,15,16]. In animal cells, ATP is primarily produced by glycolysis and oxidative phosphorylation (OXPHOS). However, glycolysis produces only two ATP per glucose molecule, whereas mitochondrial OXPHOS is more effective, providing 32 ATP [17]. If there is no glucose available, glycolysis cannot contribute to ATP production either—so the energy mainly comes from OXPHOS. In OXPHOS, the transfer of two electrons from NADH to oxygen through complexes I, III, and IV results in the translocation of ten protons across the inner mitochondrial membrane, creating the proton motive force for the synthesis of ATP by ATP synthase (complex V) [18].

While much is known about the mechanisms of MC activation and MC-driven skin symptoms, there are limited data on the activation-dependent energy metabolism of human skin MCs [19]. Most evidence on the role played by mitochondria in IgE-dependent MC activation comes from peritoneal rat MCs, murine bone marrow-derived MCs (BMMCs), the rat basophilic leukemia cell line RBL-2H3, human cord blood-derived MCs (CBMCs), and the human MC cell lines LAD2/HMC-1 [14,16,20,21,22]. Data on the mitochondrial functions in human skin MCs are almost entirely lacking. Reasons for this may include the challenging procedure of obtaining and cultivating human skin MCs and the restricted number of cells available. More insights into the energy metabolism of human skin MCs would be valuable as the skin directly faces challenging microenvironmental conditions (e.g., at sites of inflammation or hypoxia [23], wound healing [24], and in growing tumors [25]). Additionally, a better understanding of the link of mast cell activation and mitochondrial biology could help with the development of novel and better MC-targeted treatments, an important unmet need.

To analyze the functional role of the mitochondrial respiration chain in providing energy for human skin MC functions, we cultivated primary human skin MCs. Using a metabolic flux analyzer, we examined IgE-mediated activation in a minimal essential medium with amino acids and fatty acids/triglycerides, but without glucose. This approach ensures that the vast majority of ATP molecules are generated by mitochondrial OXPHOS, which allowed us to clarify the hierarchical importance of effector functions in these cells. To achieve the latter goal, we quantified the contribution of the mitochondrial complexes I and III to the performance of essential immune functions in human skin MCs (i.e., degranulation and cytokine secretion), through the use of the inhibitors rotenone A (inhibits complex I of the respiratory chain) and myxothiazol (inhibits complex III).

## 2. Results

### 2.1. IgE-Mediated Activation of Human Skin MCs Enhances Mitochondrial Respiration and ATP Coupling Efficiency, Whereas Spare Respiratory Capacity Is Reduced

**Figure 1:**
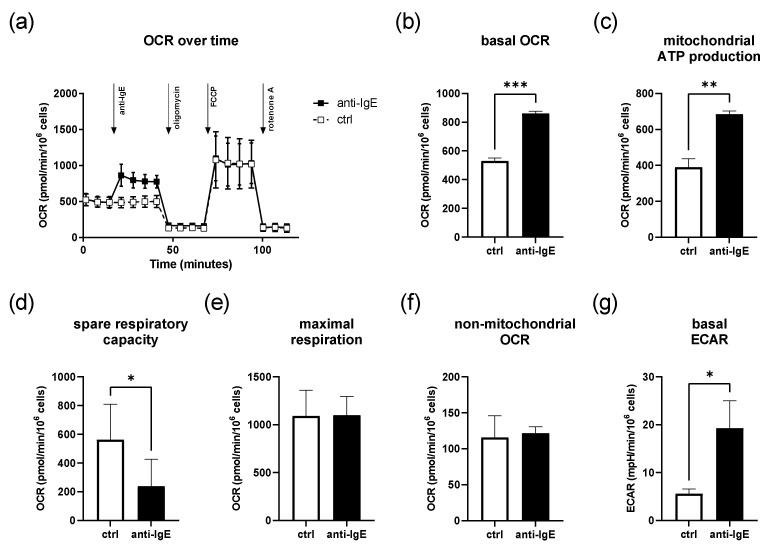
IgE-mediated activation of human skin MCs enhances mitochondrial respiration and extra-cellular acidification. (**a**–**f**) After IgE-mediated stimulation, the human skin MCs’ oxygen consumption rate (OCR) and mitochondrial parameters as well as extracellular acidification rate (ECAR) (**g**) were analyzed under glucose-free conditions in an Agilent Seahorse^TM^ metabolic flux analyzer (**a**,**g**): *n* = 3, mean ± SEM, paired Student’s *t*-test: * *p* < 0.05, ** *p* < 0.01, *** *p* < 0.001). anti-IgE = IgE-preloaded cells cross-linked with anti-IgE; ctrl = control.

First, we investigated the effects of IgE-mediated activation on the mitochondrial parameters of human skin MCs in the absence of glucose. We stimulated IgE-sensitized human skin MCs with anti-IgE and analyzed changes in the oxygen consumption rate (OCR) using the Agilent Seahorse^TM^ metabolic flux analyzer (Figure 1). A representative measurement is depicted in Figure 1a. On average, the basal OCR of the cells was 530 ± 21 pmol/min/10^6^ cells (Figure 1b). During IgE-mediated activation, the OCR increased by ~60%, and the ATP coupling efficacy was enhanced by ~75% of the control (Figure 1c). In comparison, the spare respiratory capacity (SRC) was reduced by ~43% during IgE-mediated stimulation compared to the unstimulated cells (239 ± 188 and 562 ± 247 pmol/min/10^6^ cells, respectively) (Figure 1d). Both the maximal respiration rate and non-mitochondrial OCR remained unchanged (Figure 1e,f). Despite the absence of glucose, we observed a marked increase in the basal extra-cellular acidification rate (ECAR) upon IgE-mediated stimulation (Figure 1g and Figure S1a).

### 2.2. IgE-Mediated Activation of Human Skin MCs Enhances Cytokine and Growth Factor Secretion and Degranulation

**Figure 2:**
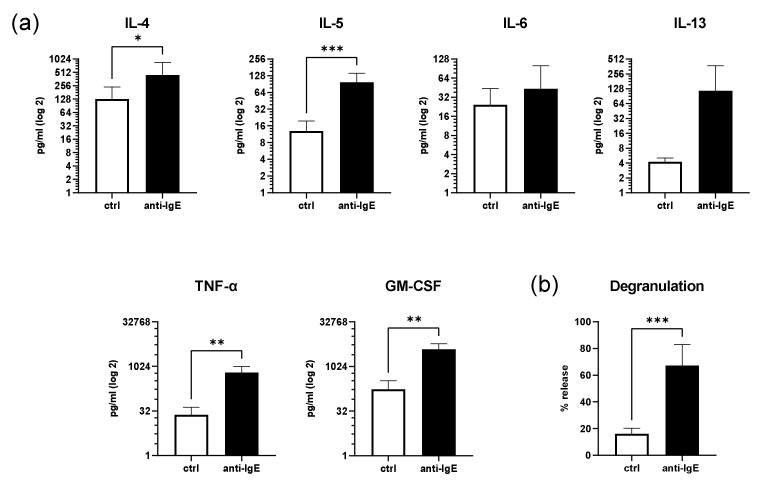
IgE-mediated activation of human skin MCs enhances cytokine and growth factor secretion and degranulation. After IgE-mediated stimulation (anti-IgE), (**a**) the human skin MCs’ cytokine and growth factor secretion (*n* = 8), and (**b**) degranulation (*n* = 7) were analyzed under glucose-free conditions. (**a**,**b**) Mean ± SEM, paired Student’s *t*-test: * *p* < 0.05, ** *p* < 0.01, *** *p* < 0.001). anti-IgE = IgE-pretreated cells cross-linked with anti-IgE; ctrl = control.

To examine the key immune functions of primary human skin MCs (i.e., degranulation and cytokine and growth factor secretion) under OXPHOS-dependent conditions, cells were loaded with human IgE and stimulated with anti-IgE in a glucose-free medium for 1 h and 16 h, respectively (Figure 2). After 16 h of IgE-mediated stimulation, the secretion of IL-4, IL-5, IL-6, IL-13, TNF-α, and GM-CSF increased, on average, by 3.5, 7.7, 1.8, 2.7, 2.7, and 2.2 times the control, respectively, which was statistically significant for the IL-4, IL-5, TNF-α, and GM-CSF levels compared to the unstimulated cells (Figure 2a). No statistically significant differences between the unstimulated and IgE-stimulated human skin MC could be detected for the secretion of IL-6 and IL-13. Furthermore, on average, the ability of degranulation remained functional under glucose-free conditions (67% ± 16, Figure 2b).

### 2.3. Inhibition of Complex III of the Respiratory Chain in Human Skin MCs Reduces Mitochondrial Parameters but Not Viability

**Figure 3:**
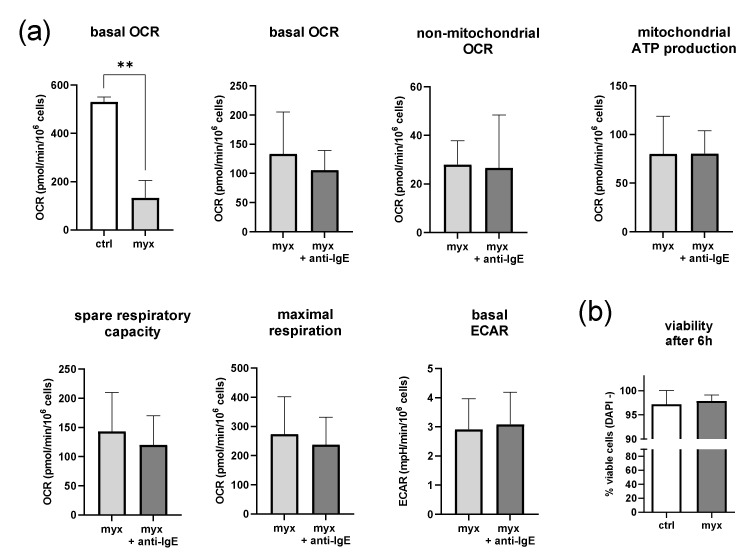
Inhibition of complex III of the respiratory chain reduces mitochondrial parameters and extracellular acidification, but not the viability of human skin MCs irrespective of IgE-mediated activation. (**a**) Inhibition of complex III of the respiratory chain in the human skin MCs using myxothiazol (myx) under glucose-free conditions reduced the mitochondrial parameters and extracellular acidification rate (ECAR). It blocked an increase in the oxygen consumption rate (OCR) after IgE-mediated activation. (**b**) Human skin MC survive (DAPI -) substantial metabolic restriction. (**a**,**b**) *n* = 3; mean ± SEM, paired Student’s *t*-test: ** *p* < 0.01), ctrl = vehicle control (1% *v/v* DMSO).

Next, we inhibited complex III of the respiratory chain in human skin MCs using myxothiazol (Figure 3). The dose of myxothiazol (10 pM) required to sufficiently decrease basal OCR (≥50% reduction) was found by titration in preliminary experiments using a Clark-type oxygen electrode (Figure S1a). On average, myxothiazol-treated human skin MCs showed a basal OCR reduced by ~75% (130 ± 67,534 pmol/min/10^6^ cells) compared to the vehicle-treated (1% *v/v* DMSO) cells (*p* < 0.01). However, no statistically significant differences in the basal OCR, non-mitochondrial OCR, ATP coupling efficacy, SRC, maximal respiration, and ECAR were detected in MCs treated with myxothiazol when comparing the unstimulated and stimulated cells (Figure 3a). Of note, ECAR was generally reduced by myxothiazol to comparable levels in the stimulated and unstimulated human skin MCs (Figure S2b,c). However, the MCs remained viable for at least 6 h in a glucose-free RPMI medium with the indicated concentration of myxothiazol (Figure 3b).

### 2.4. Inhibition of Complex III of the Respiratory Chain Does Not Affect Degranulation and Cytokine Secretion of Human Skin MCs

**Figure 4:**
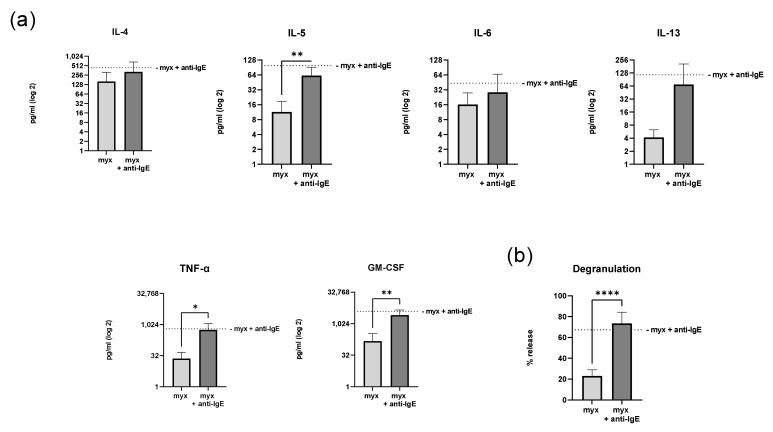
The inhibition of complex III of the respiratory chain does not affect anti-IgE-mediated cytokine and growth factor secretion and degranulation of human skin MCs. After inhibition of complex III using myxothiazol and IgE-mediated stimulation (anti-IgE), the human skin MCs’ cytokine and growth factor secretion (*n* = 8; (**a**)) and degranulation (*n* = 7; (**b**)) were analyzed under glucose-free conditions (**a**,**b**: mean ± SEM, paired Student’s *t*-test: * *p* < 0.05, ** *p* < 0.01, **** *p* < 0.0001; one sample *t*-test of myx and anti-IgE-treated human skin MCs compared to the mean value of the anti-IgE-treated control cells without myx as indicated by the dotted line). anti-IgE = IgE-preloaded cells cross-linked with anti-IgE.

To analyze the metabolic relevance of complex III within the mitochondrial respiratory chain for human skin MC activation, we assessed the cytokine and growth factor secretion and degranulation of MCs treated with myxothiazol in a glucose-free medium (Figure 4). Myxothiazol-treated human skin MCs did not differ in their IgE/anti-IgE-induced secretion of pro-inflammatory cytokines IL-4, IL-5, IL-6, IL-13, GM-CSF, and TNF-α compared to the vehicle-treated cells (Figure 4a). IgE-mediated stimulation of myxothiazol-treated MCs resulted in, on average, 2-fold higher IL-4 secretion compared to the vehicle-treated MCs, but this did not reach statistical significance. In addition, we examined degranulation using the β-hexosaminidase release assay (Figure 4b). We found that myxothiazol-treated human skin MCs did not differ in their ability to degranulate compared to the vehicle-treated cells (73% ± 11 and 67% ± 16, respectively).

### 2.5. Inhibition of Complex I of the Respiratory Chain Decreases Mitochondrial Parameters but Not Viability

**Figure 5:**
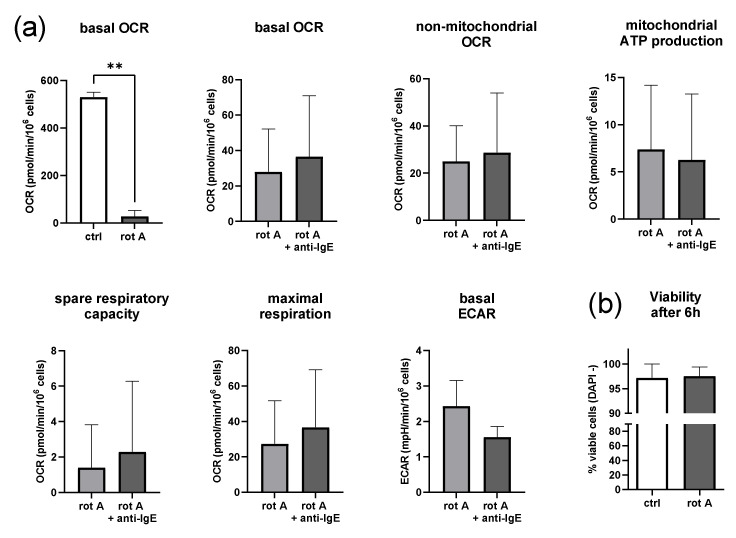
Inhibition of complex I of the respiratory chain reduces mitochondrial parameters and extracellular acidification but not the viability of human skin MCs irrespective of IgE-mediated activation. (**a**) Inhibition of complex I of the respiratory chain in human skin MCs using myxothiazol (myx) under glucose-free conditions reduced mitochondrial parameters rate (ECAR). It blocked an increase in the oxygen consumption rate (OCR) after IgE-mediated activation. (**b**) Human skin MC survive (DAPI -) substantial metabolic restriction (a and b: *n* = 3; mean ± SEM, paired Student’s *t*-test: ** *p* < 0.01), ctrl = vehicle control (1% *v/v* DMSO).

To gain a deeper understanding of the mitochondrial energy metabolism during IgE-dependent MC activation, we blocked, in the next step, complex I of the respiratory chain in human skin MCs using rotenone A (Figure 5). The amount of rotenone A required to sufficiently decrease OCR (≥50% reduction of vehicle-treated cells) was found by titration in a glucose-free medium in preliminary experiments using a Clark-type oxygen electrode (Figure S1b). The applied dose of rotenone A (5 µM) statistically significantly (*p* < 0.01) reduced OCR, on average, by 95% (28 ± 24 pmol/min/10^6^ cells) of the vehicle-treated cells (Figure 5a). No statistically significant differences in basal OCR, non-mitochondrial OCR, ATP coupling efficacy, SRC, maximal respiration, and ECAR were detected in MCs treated with rotenone A when comparing the unstimulated and stimulated cells (Figure 5a). As observed for myxothiazol, ECAR was generally reduced by rotenone A, slightly more in the stimulated than unstimulated human skin MCs, which was not statistically significant (Figure S2b,c). However, human skin MCs remained viable for at least 6 h in a glucose-free RPMI medium with the indicated amount of rotenone A (Figure 5b).

### 2.6. Inhibition of Complex I of the Respiratory Chain in Human Skin MCs Reduces Degranulation and Secretion of IL-6, IL-13, TNF-α and GM-CSF

**Figure 6:**
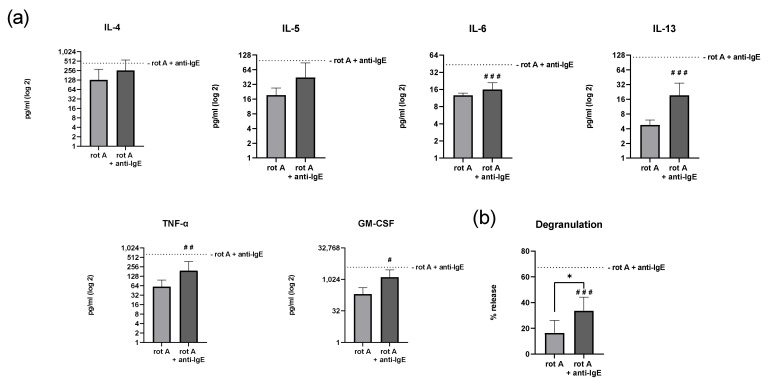
The inhibition of complex I of the respiratory chain significantly reduces degranulation and selectively attenuates anti-IgE-mediated secretion of IL-6, IL-13, TNF-α, and GM-CSF, but not of IL-4 and IL-5, of human skin MCs. After the inhibition of complex I using rotenone A (rot A), IgE-mediated stimulation (anti-IgE), the human skin MCs’ cytokine and growth factor secretion (*n* = 5; (**a**)) and degranulation (*n* = 6; (**b**)) were analyzed under glucose-free conditions (**a**,**b**: mean ± SEM, paired Student’s *t*-test: * *p* < 0.05; one sample *t*-test of rot A and anti-IgE-treated MCs compared to the mean value of anti-IgE-treated control cells without rot A as indicated by the dotted line: ^#^ *p* < 0.05, ^##^ *p* < 0.01, ^###^ *p* < 0.001). anti-IgE = IgE-pretreated cells cross-linked with anti-IgE.

Finally, human skin MCs were assessed for IgE-dependent secretion of cytokines and growth factors and degranulation after the inhibition of complex I by rotenone A (Figure 6).

Unlike the inhibition of complex III, treatment with rotenone A significantly affected the secretion of the cytokines IL-6 (16 pg/mL ± 5 vs. 44 pg/mL ± 57, *p* < 0.001), IL-13 (19 pg/mL ± 15 vs. 116 pg/mL ± 257, *p* < 0.001), TNF-α (194 pg/mL ± 186 vs. 641 pg/mL ± 370, *p* < 0.01), and GM-CSF (1316 pg/mL ± 1647 vs. 3923 pg/mL ± 2045, *p* < 0.05) (Figure 6a). No statistically significant differences in the secretion of IL-4 and IL-5 were detected. IgE-mediated degranulation remained functional in MCs treated with rotenone A (Figure 6b), but this degranulation was reduced considerably (*p* < 0.001), on average by ~50%, in cells treated with rotenone A compared to the vehicle-treated cells (34% ± 11 vs. 67% ± 16).

## 3. Discussion

This is, to the best of our knowledge, the first report on mitochondrial respiration and the role played by the respiratory chain during IgE-dependent activation in human skin MCs under metabolically compromised conditions. We chose these conditions for two reasons. First, the “battlefields” of immune cells (e.g., sites of acute and chronic tissue inflammation such as wound healing, inflamed joints, sites of ischemia, and growing tumors) are mainly characterized by diverse and often highly compromised conditions such as very low oxygen and glucose supply, low pH and increased lactate levels. Second, if glycolysis is not available for ATP production due to the lack of glucose, the hierarchical importance of effector functions in MC can be clarified in more detail.

Our results demonstrate that human skin MCs rapidly increase mitochondrial respiration and oxygen consumption after IgE-mediated degranulation and cytokine secretion without glucose. The inhibition of complex III reduced the overall OCR, but neither affected degranulation nor the secretion of cytokines. In contrast and most interestingly, complex I inhibition, which also reduced the overall OCR, markedly reduced the degranulation and secretion of IL-6, IL-13, TNF-α, and GM-CSF.

A look at the literature about the role of OXPHOS in MCs revealed that our results are partly consistent with those of others but also contradictory. On one hand, our findings align with the early observation that mitochondrial respiration is markedly increased 15–20 min post-IgE-dependent activation. It should be noted, however, that rat peritoneal MCs were used [26]. Data from the cell lines RBL-2H3 and LAD2, and BMMC demonstrate that increased mitochondrial respiration by IgE-mediated activation is STAT-3-dependent [14]. In contrast to our observations, but may be explained by the fact that cell lines were used, this study showed that a STAT-3 inhibitor that blocks complex III decreased oxygen consumption and degranulation. Additionally, pyruvate dehydrogenase (PDH), an intermediate that can regulate the tricarboxylic acid (TCA) cycle by catalyzing the conversion of pyruvate to acetyl-CoA, inhibited the mitochondrial respiration, leading to decreased IgE-mediated degranulation and reduced the secretion of TNF-α and IL-6. More recently, others have shown that IgE-dependent activation of BMMCs is associated with rapid glycolysis and not mitochondrial respiration, as the authors concluded with increased read-outs of ECAR. In line with our results, they found that BMMC degranulation and the secretion of IL-6 considerably decreased by inhibiting mitochondrial complex I with rotenone A [21].

The most obvious explanation for the differences between our results and those of the others is that all evidence on energy metabolism in MCs thus far, especially on OXPHOS, came from different types of MCs or cell lines including hematopoietic cell precursors, but not from primary human skin MCs [19]. Our results indicate that primary human skin MCs are different from other types of MCs and cell lines in several aspects. First, we found that the OCR of human skin MCs was about half as high as others have found for BMMCs [21,27], but at the same level as human monocytes and twice as high as that of human CD4+ T-cells, as shown by our recent work [28,29]. It can be assumed that OCR levels may be associated with the cell population size and mitochondrial content. Very recently, it has been shown that skin MCs associated with melanoma located in close proximity to the epidermis in the reticular layer of the dermis are smaller than MCs located in deeper layers and differ in their cytotopographic and histotopographic features [30]. Second, we observed that human skin MCs generally secrete smaller amounts of cytokines, especially IL-6, upon IgE-mediated stimulation, as described for murine MCs (i.e., BMMCs [21]). However, we found that the IgE-mediated degranulation rates of human skin MCs in the absence of glucose, first, did not differ to the rates observed at conditions with glucose, and second, were higher compared to what others have shown for BMMCs and RBL-2H3. At the same time, primary human skin MCs seem more sensitive to degranulation as they show comparable high basal degranulation rates [14,21,31]. Hence, our results suggest that the differentiation of hematopoietic MC-precursors to skin MCs includes changes in their energy metabolism and immune functions.

At present, we can only speculate why the degranulation and cytokine secretion of primary human skin MCs are selectively attenuated by the inhibition of mitochondrial complex I, but not by complex III, in the absence of glucose, even though basal OCR is substantially reduced in both instances. Myxothiazol is known to be a particular inhibitor of respiratory chain complex III. It binds to the ubiquinol oxidation site Qo of complex III and blocks electron transfer from ubiquinol to cytochrome b, inhibiting complex III activity [32,33]. Most of the energy in cells is provided via OXPHOS, and importantly, other essential ways of generating energy (i.e., glycolysis, the pentose phosphate pathway, the serine synthesis pathway, and the glycerol phosphate shuttle) were excluded in our model since they critically depend on glucose carbon. It is worth noting that ECAR significantly increased during IgE-dependent stimulation under glucose-free conditions but was found to be generally reduced by myxothiazol to comparable levels between the stimulated and unstimulated cells in our experiments. This allows for two conclusions to be drawn, first, mitochondrial respiration, but not glycolysis, makes a major contribution to ECAR in primary human skin MCs in the absence of glucose, and second, glycolysis is negligible for the maintenance of major immune functions in primary human skin MC [34].

To gain a deeper understanding of the mechanisms in the mitochondria of human skin MCs driving degranulation and cytokine secretion, we introduced rotenone A. It inhibits the transfer of electrons from iron-sulfur centers in complex I to ubiquinone and interferes with NADH when creating usable ATP. Although we found the basal OCR and ECAR was more strongly reduced by rotenone A than by myxothiazol, and the secretion of IL-4 and IL-5 was not significantly affected by these measures. Therefore, other pathways for generating energy, which are not based on glucose carbon, lactate, or OXPHOS, allow for the secretion of IL-4 and IL-5, and at least to some extent, degranulation, stressing a hierarchical importance of these immune functions based on the available energy. IL-4 and IL-5 have a wide range of immunological functions for MCs including proliferation, survival, homeostasis, induction, and the maintenance of adaptive allergic immune responses (i.e., T-helper 2 cell differentiation and induction of Ig class switch to IgE in B cells [35,36,37]). Last but not least, as fatty acid oxidation (FAO) remains another source of energy in our model, its role in degranulation and cytokine secretion (i.e., IL-4 and IL-5) needs further investigation.

Taken together, our data suggest that, on one hand, human skin MCs may actively adapt their energy metabolism to highly compromised conditions. On the other hand, the secretion of IL-4 and IL-5, and at least in part also the degranulation, but not secretion of IL-6, IL-13, TNF-α, and GM-CSF, are the favored tasks of human skin MCs when it comes to metabolically compromised conditions. Our findings may help understand the physiological functions of human skin MC at sites of hypoxic conditions, inflammation, cancer, and wound healing. Moreover, we identified critical targets of the human skin MCs’ mitochondrial electron transport chain to limit their effector function in a hierarchical manner. Further investigation of the energy metabolism in human skin MCs could contribute to a better understanding of MC physiology and their effector functions. According to our results, drugs targeting specific pathways in mitochondria [38] may provide future options to influence distinct mechanisms causing skin symptoms in MC-driven disease.

## 4. Materials and Methods

### 4.1. Preparation and Culture of Human Skin MCs

Primary human skin MCs were isolated and cultured from human breast skin (from plastic reduction surgery), eyelid, or foreskin from adult donors [39]. In brief, any adhering fat tissue was removed from the skin before cutting it into small strips. The strips were incubated overnight in PBS at 4 °C containing 2 mL dispase (50 U/mL, product number 354,235, Corning^®^, Wiesbaden, Germany). The next day, the epidermis was removed and the dermis chopped into tiny pieces, followed by enzymatic digestion using PBS containing collagenase (15 mg per gram of skin, LS004197, CellSystems, Troisdorf, Germany), hyaluronidase (7.5 mg per gram of skin, H3506, Merck, Darmstadt, Germany), and DNAse I (1 µg/mL, A3778, AppliChem, Darmstadt, Germany) at 37 °C for 1 h. After filtrating the suspension to remove any tissue residues, MCs were isolated from the cell suspension by magnetic cell sorting using the human CD117 MicroBead Kit and the Auto-MACS device (both Miltenyi Biotec, Bergisch Gladbach, Germany). Cells were cultured at 1 × 10^6^ cells/mL in Iscove’s medium, non-complete with 15 mg phenol red (FG 0465, Biochrom, Cambridge, UK) supplemented with 10% fetal bovine serum (Biochrom), 1% penicillin-streptomycin, 1% non-essential amino acids (both Thermo Fisher Scientific, Waltham, MA, USA), 250 ng/mL amphotericin B (Corning, Corning, NY, USA), and 230 µM a-monothioglycerol (Sigma-Aldrich, St. Louis, MO, USA). IL-4 (20 ng/mL, Peprotech Ref#200-04-100) and stem cell factor (SCF, 100 ng/mL, Miltenyi Ref#130-096-696) were added twice a week. The >95% purity of cultured CD117/FcεRI-positive skin MCs was routinely confirmed by flow cytometry. MCs were gated according to the size and granularity in the forward-scatter (FSC)/side-scatter (SSC) plot excluding debris. Doublets were excluded by plotting FSC-A versus FSC-H and SSC-A versus SSC-H. MCs were identified as CD117 (c-KIT), FcεRI, and X2-receptor-positive cells within the single cell gate. Gating strategy for the definition of human skin mast cells is depicted in Figure S3.

### 4.2. SeahorseTM Metabolic Flux Analysis

Metabolic assays on human skin MCs were performed using the Agilent SeahorseTM XFe96 analyzer. Human skin MCs (1 × 10^5^/well) were preloaded for 1 h with 1 µg/mL human IgE (Human IgE Myeloma, EMD Millipore) in OXPHOS dependent (glucose-free) RPMI medium (RPMI 1640, catalogue number 11879020). After a washing step, cells were left untreated or incubated with indicated concentrations of myxothiazol (inhibits complex III of the respiratory chain) or rotenone A (inhibits complex I of the respiratory chain) in SeahorseTM media for one hour. SeahorseTM media included 2 mM glutamine but no glucose in minimal, unbuffered RPMI. Then, the cells were stimulated with 1 µg/mL anti-IgE (rabbit anti-human-IgE, Bethyl Laboratories, Inc, pre-diluted 1:100), and injection of the medium served as the negative control. After 30 min of basal measurements, sequential injections of 1 mM oligomycin (inhibits mitochondrial ATP production and stimulates glycolysis), 0.5 mM FCCP (uncouples the respiratory chain and yields maximal oxygen consumption), and 0.5 mM rotenone were performed.

### 4.3. Viability Assay

Human skin MCs were incubated in a glucose-free RPMI medium in the presence of myxothiazol or rotenone. Afterward, cells were stained with DAPI (BD Pharmingen, catalogue number 559,925, dilution 1:20) according to the manufacturer’s instructions and as recently described [28]. Data were acquired using a BD FACS Canto™ II (BD Biosciences, San Jose, CA, USA) and processed by FlowJo v7.6.5 (BD Biosciences, San Jose, CA, USA). Gating strategy of the viability assay of human skin mast cells is depicted in Figure S4.

### 4.4. β-Hexosaminidase Release Assay

The β-hexosaminidase release assay to assess degranulation was performed as described previously [31]. In brief, human skin MCs (2 × 10^4^) were plated in a 96-well plate and incubated for 1 h with the indicated amounts of myxothiazol and rotenone A in Tyrode’s solution (without glucose/BSA) at 37 °C. Then, the cells were washed (300× *g*, 3 min, 22 °C) and preloaded for 1 h with 1 µg/mL human IgE in Tyrode’s solution (without glucose/BSA). After a washing step, cells were stimulated with 1 µg/mL anti-IgE (rabbit anti-human-IgE, Bethyl Laboratories, Inc., for 1 h or medium as the negative control. Cells were centrifuged (300× *g*, 3 min, 22 °C), and 50 µL of the supernatant was transferred into a black 96-well plate (Corning^®^). After discarding the remaining supernatant, cells were lysed by adding 100 µL of distilled water and frozen at −80 °C. Then, the cells were thawed, and 50 µL of the lysates were transferred to a black 96-well plate. Supernatants and cell lysates were incubated in citrate buffer (100 mM, pH 4.5) containing 4-methyl umbelliferyl-N-acetyl-beta-D-glucosaminide (4-MUG, Sigma, catalogue number M2133 working concentration 5 mM) for 1 h at 37 °C. Finally, the reaction was stopped by adding 100 µL of Na-carbonate buffer (100 mM, pH 10.7) and measured at 460 nm and excitation at 355 nm for 0.1 s using a Victor multilabel plate reader (PerkinElmer^®^, Waltham, MA, USA). The percentage of β-hexosaminidase release was calculated as: [optical density (OD) of supernatants × 100]/[OD of lysates + OD of supernatants].

### 4.5. Quantification of Cytokine Secretion

First, 1 × 10^5^ human skin MCs were incubated for 1 h with the indicated amounts of myxothiazol and rotenone A in 100 µL glucose-free RPMI (without SCF and IL-4) at 37 °C. After a washing step, cells were preloaded with 1 µg/mL human IgE in 100 µL glucose-free RPMI. Then, cells were stimulated overnight with 1 µg/mL anti-IgE or left untreated in 100 µL glucose-free RPMI. Culture supernatants from cell suspensions were obtained, and multiplex cytokine analysis was performed using the Cytokine 25-Plex Human ProcartaPlex™ Panel 1B (Thermo Fisher Scientific) according to the manufacturer’s instructions. Sample acquisition was conducted using the Bio-Plex suspension array system, and data were analyzed using the Bio-Plex Manager software (Bio-Rad, Hercules, CA, USA).

### 4.6. Statistical Analysis

Averages were presented as means ± standard deviations (SDs). Differences between two paired/dependent groups were verified using the nonparametric Wilcoxon matched pairs test. Differences between two unpaired/independent groups were demonstrated using the nonparametric Mann–Whitney test, also called the Wilcoxon rank sum test. The probability values of *p* < 0.05 were considered statistically significant. Statistical analysis was performed using GraphPad Prism 9 software.

## 5. Conclusions

Our study demonstrates that complex I of the mitochondrial chain, and not complex III, is essential for the performance of degranulation and cytokine secretion by primary human skin MCs during metabolically compromised conditions. The inhibition of complex I attenuated IgE-mediated secretion of IL-6, IL-13, TNF-α, and GM-CSF and, at least to some extent, degranulation, but not the secretion of IL-4 and IL-5, while OCR and ECAR were found to be reduced. These results demonstrate the complex energy metabolism of primary human skin MCs and allow us to conclude on a hierarchy of their immune functions on the basis of available energy.

## Data Availability

The data presented in this study are available on request from the corresponding author. The data are not publicly available due to privacy and ethical restrictions.

## Supplementary Materials

The following supporting information can be downloaded at: https://www.mdpi.com/article/10.3390/ijms231911591/s1.
