# Supplementary material for: Inhibition of Complex I of the Respiratory Chain, but Not Complex III, Attenuates Degranulation and Cytokine Secretion in Human Skin Mast Cells
Source: Int J Mol Sci. 2022 Sep 30;23(19):11591. doi: 10.3390/ijms231911591 (PMC9570238; doi:10.3390/ijms231911591)
Supplement: Supplementary file 1 [file ijms-23-11591-s001.zip › ijms-1919674-supplementary.pdf]

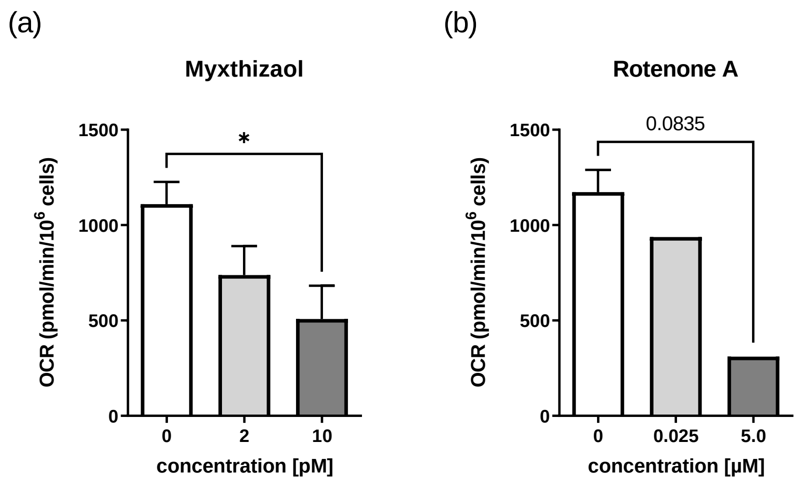

Figure S1. Titration of myxothiazol and rotenone A in human skin MCs in a glucose-free medium. Oxygen consumption rate (OCR) of human skin MCs was measured in a glucose-free RPMI medium in the presence of different concentrations of (**a**) myxothiazol (n = 3-6; [pM]) or (**b**) rotenone A (n = 2-6; [µM]) or 1% (v/v) DMSO (vehicle control at 0 pM) using a Clark-type electrode (mean ± SEM; ordinary one-way ANOVA and Tukey's multiple comparison test, with a single pooled variance, , * $p < 0.05$).

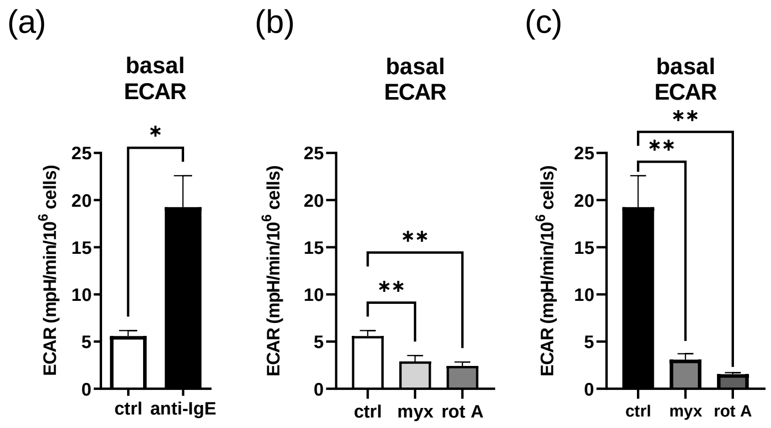

Figure S2. Impact of myxothiazol and rotenone A on extracellular acidification rate (ECAR) in resting and anti-IgE stimulated human skin MCs in a glucose-free medium. Extracellular acidification rate (ECAR) of human skin MCs was measured in resting (**a** and **b)** and in anti-IgE-stimulated cells (**a** and **c**) in the absence (**a**) and presence (**b** and **c**) of either myxothiazol (myx) [10pM] or rotenone A (rot A) [5µM]) under glucose-free conditions in the Agilent Seahorse^{TM} metabolic flux analyser (a-c: n=3; mean ± SEM; a: paired Student's t-test: * $p < 0.05$; b and c: repeated measures one-way ANOVA and Tukey's multiple comparison test, ** $p < 0{,}01$ with a single pooled variance); ctrl = control.

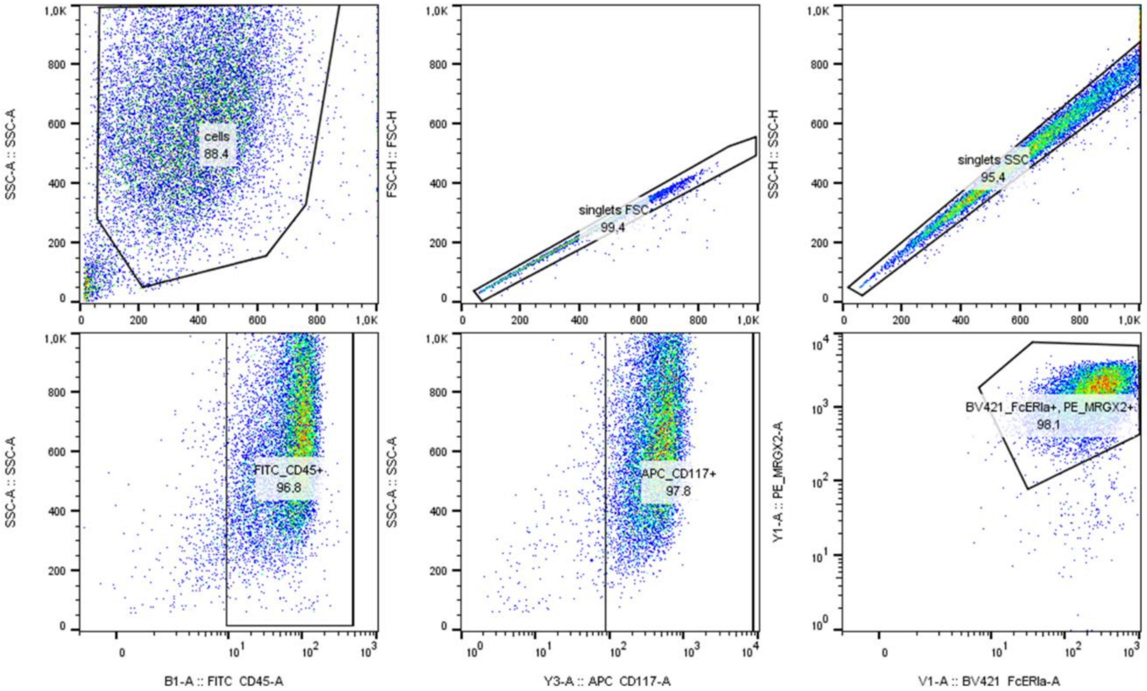

Figure S3. Gating strategy for the definition of human skin mast cells.

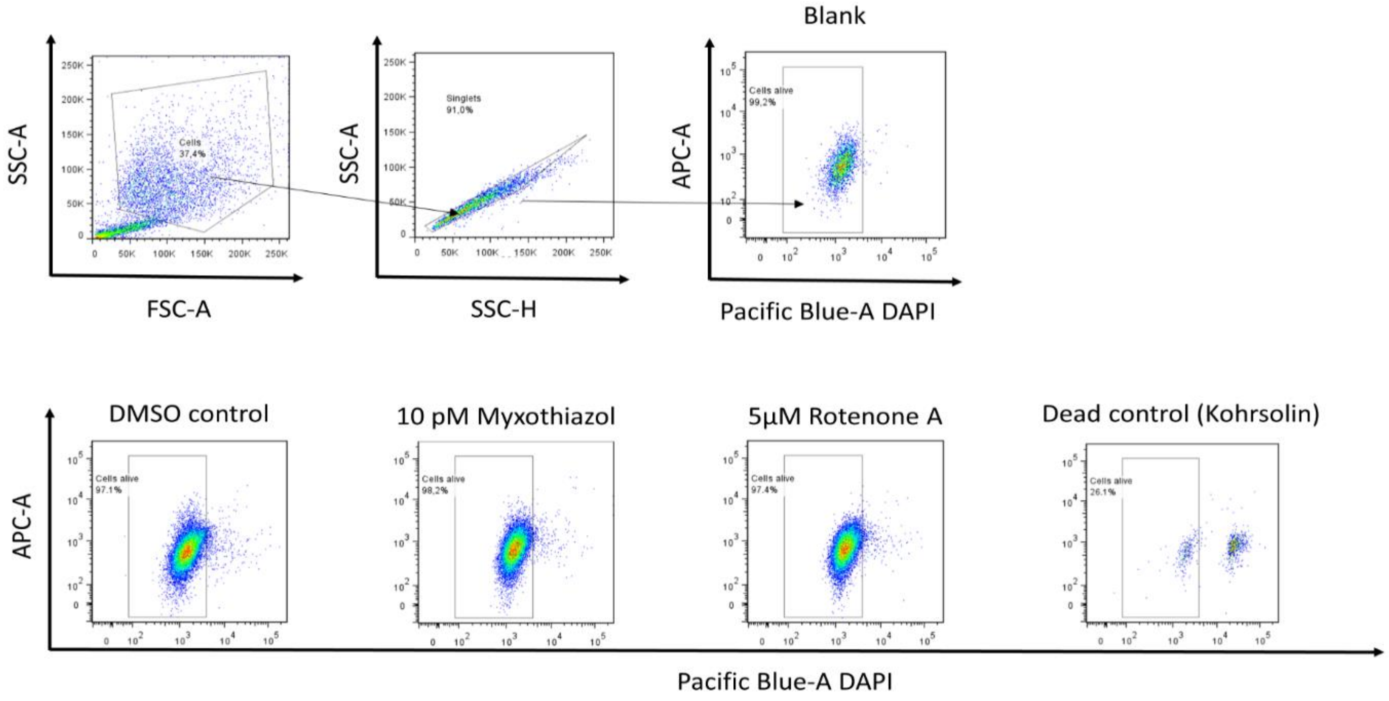

Figure S4. Gating strategy of viability assay of human skin mast cells.
